# Single‐Cell Transcriptomics Revealed E‐Cigarettes‐Induced Vascular Remodeling by Enhancing *Tcf21* Expression

**DOI:** 10.1002/mco2.70183

**Published:** 2025-04-17

**Authors:** Ruiyang Ding, Xiaoke Ren, Qinglin Sun, Shiqian liu, Linyuan Huang, Zhiwei Sun, Junchao Duan

**Affiliations:** ^1^ Department of Toxicology and Sanitary Chemistry School of Public Health Capital Medical University Beijing People's Republic of China; ^2^ Laboratory for Clinical Medicine Capital Medical University Beijing People's Republic of China

**Keywords:** electronic cigarette, single‐cell sequencing, vaping, vascular remodeling

1

Dear Editor,

Electronic cigarettes (e‐cigs) are battery‐powered new tobacco products attracting wide attention due to their potential to promote smoking cessation and uncertainty in long‐term health effects. The global market of e‐cigs is still expanding with a compound annual growth rate of 30.6% from 2023 to 2030, and the United States has the largest e‐cig market with an e‐cig prevalence in 4.5% of adults and 10% of high school students [1, 2]. Although e‐cigs were generally considered safer than canonical tobacco products, the outbreak of vaping‐associated lung injury in 2020, which incurred more than 2800 hospital admissions, highlighted their potential threat to human health. According to the scientific statement from the American Heart Association (AHA), e‐cig use may impose a latent risk to the cardiopulmonary system, and epidemiological studies suggested that e‐cig aerosol could enhance arterial stiffness in humans [3]. Laboratory evidence suggested that e‐cigs could accelerate atherosclerosis and aortic aneurysms in mice [4, 5], while the exact mechanisms of e‐cig‐related effects on the vasculature remained largely unknown. Here, we investigate the sub‐chronic effects of e‐cig aerosol on vascular structure, function, and changes in single‐cell transcriptome in mice.

C57BL/6J male mice aged 8 weeks inhaled filtered air (as control) or e‐cig (RELX, containing glycerin, propylene glycol, spices, and 3% nicotine) 1 h per day in the exposure chamber for consecutive 90 days (Figure 1A, upper panel). Details about exposure patterns were provided in the Supplementary Information. Hematoxylin and eosin (H&E) staining indicated that sub‐chronic exposure to e‐cig aerosol increased the media thickness and promoted the vascular smooth muscle cell (VSMC) hyperplasia and disordered arrangement (Figure 1A, lower panel). Verhoeff's Van Gieson (EVG) staining revealed the fractured and distorted distribution of the elastic fibers in mice exposed to e‐cig aerosol. Doppler ultrasound assessment indicated that e‐cig aerosol exposure reduced the left common carotid artery pulsatility index (LCCA PI) while enhancing the carotid intima‐media thickness (CIMT), pulse wave velocity (PWV), left common carotid artery peak systolic velocity (LCCA PSV), and left common carotid artery end‐diastolic velocity (LCCA EDV). These results suggested that long‐term exposure to e‐cig aerosol increased the aortic wall thickness and impaired arterial function.

**FIGURE 1:**
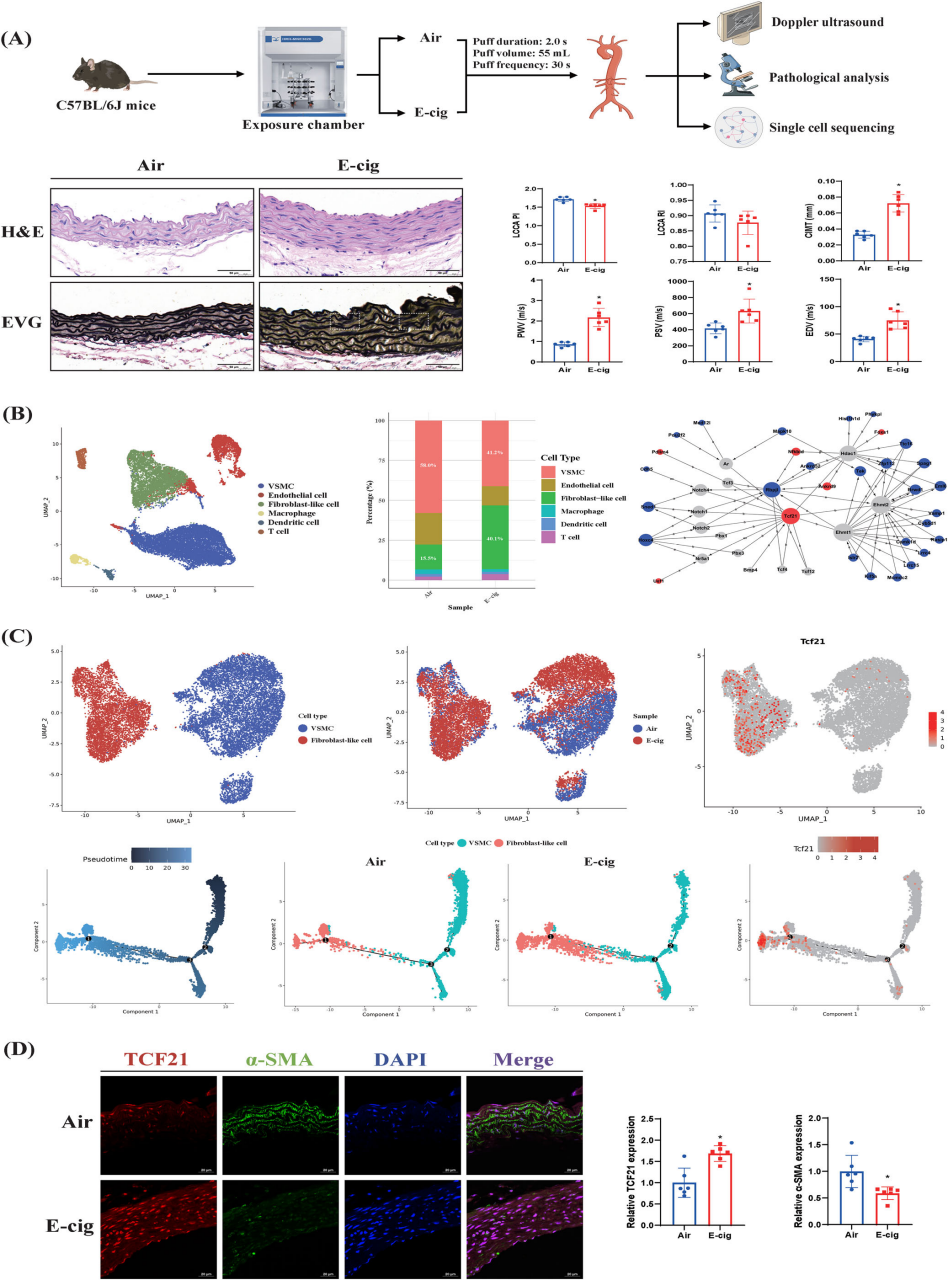
C57BL/6J mice were exposed to either filtered air or electronic cigarette (e‐cig) aerosol in the exposure chamber. The results were expressed as means ± standard deviations, and statistical significance was evaluated by the student's *t*‐test in the GraphPad Prism 8. *Represented statistically significant (*p *< 0.05) in mice exposed to e‐cig aerosol compared to mice exposed to filtered air. (A) Schematic illustration of the exposure procedure (upper panel). Hematoxylin and eosin (H&E) staining of pathological changes and Verhoeff's van Gieson (EVG) staining of elastic fibers in the aorta of mice, with white dashed boxes indicating fragmented and disorganized elastic fibers (lower panel). Doppler ultrasound assessment of the left common carotid artery pulsatility index (LCCA PI), left common carotid artery resistant index (LCCA RI), the carotid intima‐media thickness (CIMT), pulse wave velocity (PWV), left common carotid artery peak systolic velocity (LCCA PSV), and left common carotid artery end‐diastolic velocity (LCCA EDV) (*n* = 6). (B) Uniform Manifold Approximation and Projection (UMAP) visualization of cell clusters (left panel). Counting proportions of vascular smooth muscle cells (VSMCs) and fibroblast‐like cells from different samples (middle panel). Network analysis of differentially expressed genes in both VSMCs and fibroblasts. Red represents significantly upregulated genes, blue represents significantly downregulated genes, and gray represents connecting genes that do not exhibit significant changes themselves but may be involved in signal transduction (right panel). (C) UMAP map of VSMCs and fibroblast‐like cells with annotation of *Tcf21* expression (upper panel). Pseudotime analysis of VSMCs and fibroblast‐like cells (lower panel). Different colors of latent time represent different differentiation times, with darker shades of blue indicating earlier times and lighter shades indicating later times. VSMCs and fibroblast‐like cells from different samples were also annotated with green (VSMCs) and red (fibroblast‐like cells). The expression of Tcf21 was also presented along the pseudotime trajectory. (D) Immunofluorescence assay of TCF21 and α‐smooth muscle actin (α‐SMA) in mouse aorta (*n* = 6).

The whole aorta samples collected from mice were used for single‐cell RNA transcriptome sequencing (scRNA‐seq), and the raw data was deposited to the Gene Expression Omnibus (GEO), accession number GSE288003. scRNA‐seq identified 20 cell clusters in the aorta samples from mice exposed to filtered air or e‐cig aerosol, and they could be classified as six cell lineages according to specific markers, including VSMC, endothelial cell, fibroblast‐like cell, macrophage, dendritic cell, and T cell (Figure 1B, left panel). After counting the percentage of cell population in different samples (Figure 1B, middle panel), we observed a relative increase in the percentage of fibroblast‐like cells following e‐cig exposure (40.1% vs. 15.5%) and a reduction in the VSMC population (41.2% vs. 58.0%). VSMCs are important regulators of vascular function, as they directly formulate the tunica media and maintain vascular strength and elasticity. Under pathological stimulation, VSMCs can switch to a fibrotic phenotype to promote extracellular matrix (ECM) deposition, which is a key process in vascular remodeling [6]. Therefore, we speculated that the increased number of fibroblast‐like cells may result from phenotype switching of VSMCs. To investigate the underlying mechanisms, we then obtained an intersection of differentially expressed genes induced by e‐cig exposure in both VSMCs and fibroblast‐like cells. As shown in Figure 1B (right panel), the network of these genes indicated that *Tcf21* may be an essential regulator in genes altered by e‐cig exposure, and it was upregulated in both VSMCs (FC = 2.83, *p* = 0.03) and fibroblast‐like cells (FC = 2.69, *p* < 0.001).

To confirm the role of *Tcf21*, we searched for its role in published literature from the PubMed database. Interestingly, *Tcf21* was recently reported to suppress the expression of contractile‐related genes, including *TAGLN*, *ACTA2*, and *MYH11*, whereas enhanced genes related to phenotypic modulation, such as *Col1α1*, *Tnfrsf11b*, and *FN1*, resulted in a transformation into fibroblast‐like cells [7, 8]. *Tcf21* is minimally expressed in VSMCs under the contractile phenotype, while highly expressed in fibroblasts and VSMC‐derived ‘fibromyocytes’ [9]. We conducted a more focused annotation of VSMCs and fibroblast‐like cells in Figure 1C, and it was revealed that most fibroblast‐like cells were from the e‐cig exposed group. The Uniform Manifold Approximation and Projection (UMAP) visualization of *Tcf21* also revealed that this gene was mostly expressed in fibroblast‐like cells but also showed increased expression in a small subset of VSMCs, which may be attributed to e‐cig exposure (Figure 1C, upper panel). Moreover, the pseudotime analysis of these two cell types exhibited three main branches and one main trajectory (Figure 1C, lower panel). Along the pseudotime trajectory, cells at the early stage of differentiation were predominantly VSMCs and mainly derived from the control group, while cells at the late stage of differentiation were almost entirely fibroblast‐like cells and primarily from the e‐cig exposure group. Besides, *Tcf21* was also predominantly localized at the end of the pseudotime trajectory, as it was expressed in a minor subset of VSMCs but highly enriched in fibroblast‐like cells. These observations further supported that e‐cig exposure may contribute to vascular remodeling by promoting the differentiation of VSMCs into fibroblast‐like cells. As *Tcf21* was reported to inhibit the expression of *ACTA2*, which encoded α‐smooth muscle actin (α‐SMA) in VSMCs, an immunofluorescence assay was conducted to verify the protein expression of TCF21 and α‐SMA in aortic tissues. The results indicated that the expression of TCF21 increased in mice exposed to e‐cigs, which was accompanied by reduced expression of α‐SMA (Figure 1D), suggesting a potential transformation from contractile to synthetic phenotype [6, 7].

TCF21 was initially identified as a causal gene for coronary artery disease (CAD) but was first reported to regulate the phenotype switching of VSMCs to fibroblast‐like cells in atherosclerotic lesions of both mice and human samples [8]. Under the pathological stimulation, TCF21 served as a key regulator in promoting the differentiation of VSMCs into a synthetic phenotype that produced ECM, as well as facilitating their proliferation and migration into atherosclerotic lesions to form a fibrous cap [7]. Therefore, it was speculated that increased fibroblast‐like cells in the e‐cig group may result from phenotype switching of VSMCs driven by *Tcf21*. Overexpression of *Tcf21* was reported to inhibit the expression of contractile markers such as smooth muscle protein 22α (SM22α) and α‐SMA in VSMCs, whereas it enhanced the expression of inflammatory cytokines, including IL‐1β and IL‐6 [10]. Moreover, *Tcf21* was also found to regulate ECM secretion and cellular adhesion in VSMCs, implicating that e‐cigs may alter VSMC function and incur subsequent vascular remodeling through enhancing *Tcf21* expression [9]. To the best of our knowledge, our study was the first to adopt single‐cell sequencing on the aorta of mice exposed to e‐cigs, which provided a basis for future studies to elucidate the mechanisms of e‐cig‐related vascular effects. However, this study has certain limitations. Firstly, there is still a lack of in vitro models to confirm the role of *Tcf21* in e‐cig‐related VSMC phenotype switching, as e‐cigs impose a complex effect on the vasculature after inhalation and cannot be directly simulated in cultured cell lines. Moreover, scRNA‐seq in this study was based on a mouse model; correlated gene alteration should be further verified in human samples, particularly e‐cig users. Therefore, future studies were still required to investigate more significant outcomes after a prolonged exposure period and elucidate the role of *Tcf21* in e‐cig‐related adverse effects.

## Author Contributions

**R.D**.: formal analysis, investigation, visualization, data curation, writing – original draft. **X.R**.: methodology, validation. **Q.S**.: methodology, validation. **S.L**.: methodology, validation. **L.H**.: methodology, validation, data curation. **Z.S**.: resources, supervision, writing – review and editing. **J.D**.: conceptualization, supervision, writing – review and editing. All authors have read and approved the final manuscript.

## Ethics Statement

All animal studies were approved by the Capital Medical University (ethical review number: AEEI‐2021‐024).

## Conflicts of Interest

The authors declare no conflicts of interest.

## Supporting information



Supporting information

## Data Availability

The scRNA‐seq data was deposited to the Gene Expression Omnibus (GEO), accession number GSE288003.
