# Supplementary material for: Single‐Cell Transcriptomics Revealed E‐Cigarettes‐Induced Vascular Remodeling by Enhancing Tcf21 Expression
Source: MedComm (2020). 2025 Apr 17;6(5):e70183. doi: 10.1002/mco2.70183 (PMC12004124; doi:10.1002/mco2.70183)
Supplement: Supplementary file 1 — Supporting information [file MCO2-6-e70183-s001.pdf]

Supplementary Information

Single-cell transcriptomics revealed e-cigarettes-induced vascular remodeling by enhancing Tcf21 expression

Ruiyang Ding [1,2], Xiaoke Ren [1,2], Qinglin Sun [1,2], Shiqian liu [1,2], Linyuan Huang [1,2], Zhiwei Sun [1,2], Junchao Duan [1,2,*]

[1] *Department of Toxicology and Sanitary Chemistry, School of Public Health, Capital Medical University, Beijing 100069, P.R. China*

[2] *Laboratory for Clinical Medicine, Capital Medical University, Beijing 100069, P.R. China*

Materials and methods

Animal treatment

All animal studies were approved by the Capital Medical University (ethical review number: AEEI-2021-024). C57BL/6J male mice aged 8 weeks inhaled filtered air (as control) or e-cig (RELX, containing glycerin, propylene glycol, spices, and 3% nicotine) 1 h per day in the exposure chamber for consecutive 90 days. The exposure duration was selected based on a previously published study by our group, which indicated that acute exposure to e-cigs for 1 h could induce metabolomic alterations, oxidative stress, and apoptosis in the lungs and hearts of mice [1]. Besides, we also referred to other published studies of e-cigs, which set the exposure durations ranged from 1-3 h/day for 3-6 months [2-5]. The smoking pattern was established primarily based on ISO 3308 and modified according to the World Health Organization (WHO) guiding principles, briefly as follows: 2.0 s puff duration, 55 mL puff volume, 30 s puff frequency [1]. The vascular Doppler ultrasound assessment was conducted with the ultra-high resolution color Doppler ultrasound imaging system (Vevo 2100 Imaging System, FUJIFILM VisualSonics Inc., USA) after the exposure procedure, as described in our previous studies [2]. All mice were anesthetized with tribromoethyl ether and sacrificed together, then the aortic samples were collected for subsequent assays.

Single-cell transcriptomics

The whole aorta samples (n=3 in each group) collected from mice were used for single-cell transcriptome. Due to the large tissue input required for single-cell sequencing, samples in each group were polling together before the transcriptomics. Tissues were transported to sterile culture dish with 10 ml 1x Dulbecco's Phosphate-Buffered Saline (DPBS; Gibco, Cat. no. 14200075) on ice to remove the residual tissue storage solution, then minced on ice. We used 0.25% Trypsin (Sigma, Cat. no. T6414) and 10 ug/mL lDNase I (Sigma, Cat. no. DN25) dissolved in PBS with 5% Fetal Bovine Serum (FBS; Thermo Fisher, Cat. no. SV30087.02)

to digest the tissues. Testis were dissociated at 37 °C with a shaking speed of 50 r.p.m for about 40 min. We repeatedly collected the dissociated cells at interval of 20 min to increase cell yield and viability. Cell suspensions were filtered with a 40 µm nylon cell strainer and red blood cells were removed by 1X Red Blood Cell Lysis Solution (Solarbio Cat. No. R1010). Dissociated cells were washed with 1x DPBS containing 2% FBS, then stained with AO/PI to check the viability on the Countstar Fluorescence Cell Analyzer (Countstar).

scRNA-seq libraries were constructed using the 10x Genomics Single Cell Gene Expression Solution v3.1. All the procedures were performed according to the standard manufacturer's protocol (CG000315 Rev E). Briefly, beads with unique molecular identifier (UMI) and cell barcodes were loaded close to saturation, so that each cell was paired with a bead in a Gel Beads-in-emulsion (GEM). After exposure to cell lysis buffer, polyadenylated RNA molecules hybridized to the beads. Beads were retrieved into a single tube for reverse transcription. On cDNA synthesis, each cDNA molecule was tagged on the 3'end with UMI and cell label, indicating its cell of origin. Then cDNA along with cell barcodes were PCR-amplified.The constructed libraries were quantified using a High Sensitivity DNA Chip (Agilent) on a Bioanalyzer 4150 and the Qubit High Sensitivity DNA Assay (Thermo Fisher Scientific). Then the libraries were sequenced on NovaSeq 6000 (Illumina) with a sequencing depth of at least 20,000 reads per cell with a pair-end 150 bp (PE150) reading strategy. Markers for cell type annotations were as follows: VSMC (*Myh11, Tagl*, *Acta2*), fibroblast-like cell (*Col1a1*, *Col3a*, *Dcn*), endothelial cell (*Pecam1*, *Cdh5*, *Fabp4*), macrophage (*Cd68*, *C1qb*, *Mrc1*), T-cell (*Cd3d*, *Cd3g*, *Cd28*), and dendritic cell (*Ccr7*, *Cd74*, *H2-Eb1*) [6-8].

For network analysis, we first obtained the intersection of differentially expressed genes in vascular smooth muscle cells (VSMCs) and fibroblasts after e-cig exposure, and then the relationship between each gene was built based on Kyoto Encyclopedia of Genes and Genomes (KEGG) and String database. With the help of the pathway topology in databases,

significantly changed pathways were connected in a network to show the relationship between genes involved in these pathways. The visualization of network was built by software Cytoscape (vension:3.6.0).

Pseudotime analysis was performed to reconstruct cellular developmental trajectories using the Monocle 3 algorithm. Single-cell transcriptomes were projected into a reduced-dimensional space via UMAP (Uniform Manifold Approximation and Projection) with the top 2,000 highly variable genes selected by variance-stabilizing transformation (VST). Cell ordering along pseudotime was determined using the reversed graph embedding approach, which models transcriptional dynamics as a principal graph embedded in the low-dimensional space. Branch points in the trajectory were identified through partition-based graph abstraction (PAGA) to delineate divergent differentiation paths.

Histopathological analysis

After euthanasia by cervical dislocation, the aortic tissue was harvested from the mice. Subsequently, segments of the thoracic aorta were preserved in 4% paraformaldehyde for 24 hours, facilitating histopathological examination. These aorta segments were then embedded in paraffin and sliced into 5-micrometer-thick sections. To visualize histopathological changes and elastic fibers, the prepared paraffin sections were stained using both hematoxylin and eosin (H&E) and Verhoeff's van Gieson (EVG) stains. The slices were imaged by automatic slice scanning system (3Dhistech, Hungary).

Immunofluorescence

Prior to antibody treatment, the paraffin sections underwent dewaxing and rehydration processes. They were then incubated with primary antibodies including anti-alpha smooth muscle actin (ab7817, Abcam) and TCF21 antibody (DF13477, Affinity) at 4 °C overnight. Following this, all samples were incubated with correlated secondary antibody (#4412 (Anti-rabbit IgG) and #5470 (Anti-mouse IgG), Cell Signaling Technology) at 37°C for an hour.

Subsequently, they were stained with DAPI (H-1200-10, VECTASHIELD® Antifade Mounting Medium with DAPI) in the dark for 10 minutes. The preparations were examined under a laser confocal microscope and analyzed using the Image J software.

References

1. Ren X, Lin L, Sun Q, et al. Metabolomics-based safety evaluation of acute exposure to electronic cigarettes in mice. *Sci Total Environ*. Sep 15 2022;839:156392. doi:10.1016/j.scitotenv.2022.156392
2. Ning R, Li Y, Du Z, et al. The mitochondria-targeted antioxidant MitoQ attenuated PM(2.5)-induced vascular fibrosis via regulating mitophagy. *Redox biology*. Oct 2021;46:102113. doi:10.1016/j.redox.2021.102113
3. Wong ET, Szostak J, Titz B, et al. A 6-month inhalation toxicology study in Apoe(-/-) mice demonstrates substantially lower effects of e-vapor aerosol compared with cigarette smoke in the respiratory tract. *Arch Toxicol*. May 2021;95(5):1805-1829. doi:10.1007/s00204-021-03020-4
4. Reumann MK, Schaefer J, Titz B, et al. E-vapor aerosols do not compromise bone integrity relative to cigarette smoke after 6-month inhalation in an ApoE(-/-) mouse model. *Arch Toxicol*. Jun 2020;94(6):2163-2177. doi:10.1007/s00204-020-02769-4
5. Maishan M, Sarma A, Chun LF, et al. Aerosolized nicotine from e-cigarettes alters gene expression, increases lung protein permeability, and impairs viral clearance in murine influenza infection. *Front Immunol*. 2023;14:1076772. doi:10.3389/fimmu.2023.1076772
6. Zhao G, Lu H, Chang Z, et al. Single-cell RNA sequencing reveals the cellular heterogeneity of aneurysmal infrarenal abdominal aorta. *Cardiovasc Res*. Apr 23 2021;117(5):1402-1416. doi:10.1093/cvr/cvaa214
7. Cochain C, Vafadarnejad E, Arampatzi P, et al. Single-Cell RNA-Seq Reveals the Transcriptional Landscape and Heterogeneity of Aortic Macrophages in Murine Atherosclerosis. *Circulation research*. Jun 8 2018;122(12):1661-1674. doi:10.1161/circresaha.117.312509
8. Wirka RC, Wagh D, Paik DT, et al. Atheroprotective roles of smooth muscle cell phenotypic modulation and the TCF21 disease gene as revealed by single-cell analysis. *Nat Med*. Aug 2019;25(8):1280-1289. doi:10.1038/s41591-019-0512-5
